# Crisis-Like Behavior in China's Stock Market and Its Interpretation

**DOI:** 10.1371/journal.pone.0117209

**Published:** 2015-02-06

**Authors:** Fangli Fan, Jianbo Gao, Shuhong Liang

**Affiliations:** 1 Institute of Complexity Science and Big Data Technology, Guangxi University, Nanning, Guangxi, P. R. China; 2 Business School, Guangxi University, Nanning, Guangxi, P. R. China; 3 PMB Intelligence LLC, Sunnyvale, United States of America; Universidad Veracruzana, MEXICO

## Abstract

In order for China to play a bigger, more positive role in the world, it is important for China to have a healthy capital market. This perception motivates us to examine the health of China's capital market, especially the severity of the overall loss of the listed companies in China and the effects of accounting irregularities on the losses. We show the overall loss of the listed companies was very severe, in particular, crisis-like behavior emerged in the fourth quarter of 2002, 2004, 2005, and 2008. We further observe that loss in the fourth quarter was much greater than the average loss of the first three quarters in the same year. The most straightforward interpretation of this loss pattern is that companies underreported losses in the first three quarters, to boost their stock values in most time of the year. However, in the fourth quarter, accounting balance of the whole year dictated that the reported loss in the fourth quarter had to be much greater than the actual loss. Fortunately, such irregularity has been greatly reduced, thanks to the accounting reforms in China in 2007.

## Introduction

As the world's second largest economy, China has been playing an ever increasing role in the world's politics, economics, culture, foreign policy, international trade, military, among others. In particular, China has played a critical role in helping the rest of the world to recover from the recent gigantic Financial Crisis. To the great benefit of China and the world, it is critical for China to have a healthy market economy. One important component of the market economy is the capital markets.

As is well-known, the level of development of capital markets is closely related to the degree of economic development of a country. Healthy capital markets can provide an effective financing platform for the enterprise. Through an IPO financing, listed companies can develop better and faster. In economically developed countries, the earnings of a large number of listed companies often can well reflect the economy of the country. However, in developing economies, the capital market is yet to evolve to be perfect, and the weight of listed companies in the national economy is small, therefore, the status of their development cannot fully reflect the macroeconomy of the country [1]. However, recent studies have shown that as the economy continues to develop, there is not only a correlation between the macroeconomy and capital markets, but that the influence of capital markets on macroeconomy is increasing [2]. Therefore, study of the earnings of listed companies in a developing economy can still help understand the economy of the country. Based on this rationale, many researchers have devoted their attention to the study of the earnings of the Chinese A-share listed companies. So far, published researches have focused on accounting cheating [3], tunneling behavior [4, 5], profit manipulation [6], and identification of these abnormal behaviors [7]. These studies are largely qualitative; albeit some of them have studied certain specific factors underlying a phenomenon, they have ignored some of the more fundamental issues, such as how severe is the overall loss of the listed companies? are the losses of the listed companies abnormal? is the accounting practice associated with these losses consistent with established norms? do the losses harm the interests of investors? Further accompanying these issues are (1) are the abnormal behaviors decreasing? and (2) is China's capital market becoming healthier?

An extreme case of loss is generated by financial crises. In the past three decades, the world has seen many countries experiencing financial crises with different degrees of severity. Especially costly is the 2008 global financial crisis, which has affected essentially all the industrialized countries, as well as a large number of developing economies. The grave cost of the crisis has pushed early warning system (EWS) models for predicting and mitigating severity of future crises into the spotlight.

Existing EWS models include the leading indicator approach [8], the discrete-dependent-variable approach based on logit and probit models [9, 10], structural equation model [11], and network-based models [12, 13]. The results obtained so far are mixed, however. For example, the loss of information using a binary variable in the leading indicator approach is questionable [14–16]. After carefully examining the Multiple-Indicator Multiple Cause (MIMIC) model of Goldberger [11], Rose and Spiegel [17] in particular, have concluded that few of the characteristics suggested as potential causes of the crisis actually help predict the intensity and severity of the crisis across countries. The best indicators for the 2008 crisis include asset price inflation, rising leverage, large sustained current account deficits, and a slowing trajectory of economic growth [18]. Overall, however, economists have not had a particularly good track record at predicting the timing of crises [17]. This has motivated us to develop an entropy-based indicator of financial crisis [19, 20].

To gain insights into the numerous important questions we have posed about the health of China's capital markets, we examine the behaviors of companies listed in China's A-share market. While a company's activity is characterized by many variables, the most important one has to be the earning, or the profit. Therefore, we will apply the entropy approach of Gao et al. [19, 20] to examine the earnings of thousands of Chinese A-share listed companies.

## Materials and Methods

### 1 Data

We examine 2468 Chinese A-share listed companies' profit data. These data are downloaded from the Wind database (http://www.wind.com.cn/). These companies are divided into 12 sectors including AFLF (agriculture, forestry, livestock, and fishery), Construction, Culture, Electricity, Extractive, Financial, Information Technology (IT), Manufacturing, Real estate, Retail, Services, and Transportation.

Due to absence of accounting audit in Q1-Q3 in China, it is generally thought that yearly total profit data are more meaningful than quarterly profits. To best reveal the working mechanism of accounting and other irregularities, we will focus on quarterly profits in this work. By computing entropies for positive and negative profits of companies in different sectors at different times, we show that there exist economic recession or financial crisis-like behaviors in many sectors of China's economy. Such behaviors exclusively occurred in the fourth quarter of a number of years. Accompanying this were the abnormally low Q4's earnings. Such behaviors were clearly not consistent with a good capital market. Fortunately, such irregularity has been greatly reduced, thanks to accounting reforms in China in 2007.

### 2 Shannon entropy

The stability or strength of an profit cluster may be quantified by its entropy, given that the second law of thermodynamics can be equivalently restated as saying that the most stable configuration is the one with the highest entropy [21]. For the negative and positive profit clusters considered here, the Shannon entropy is a pertinent measure, and for discrete probabilities is given by [22]:

$$H=-\sum P_{i}\log P_{i},$$ (1)

where *P*_*i*_ are the probabilities that the positive or negative profits will fall within a prescribed bin *i* (where a bin is an interval of fixed length). We shall take 2 as the base of the logarithm so that the unit of the entropy is the bit. Note that when all the probabilities are equal, Shannon entropy attains its largest value; such a situation may be associated with the discretization of a uniform distribution.

### 3 Distributional analysis

When examining the distribution of losses due to financial crises, Gao et al. [19, 20] have found that the most pertinent distribution is the heavy-tailed distribution, which includes the celebrated Pareto-law as a special case:

$$P(X\geq x)∼x^{-\alpha },x\to \infty$$ (2)

where α is a parameter. When 0 < α < 2, the distribution has infinite variance, and when 0 < α ≤1, the mean is also infinite [23]. This is the distribution that we will focus on in the later discussions.

## Results

### 1 Crisis-like behavior in China's capital market

**Fig 1:**
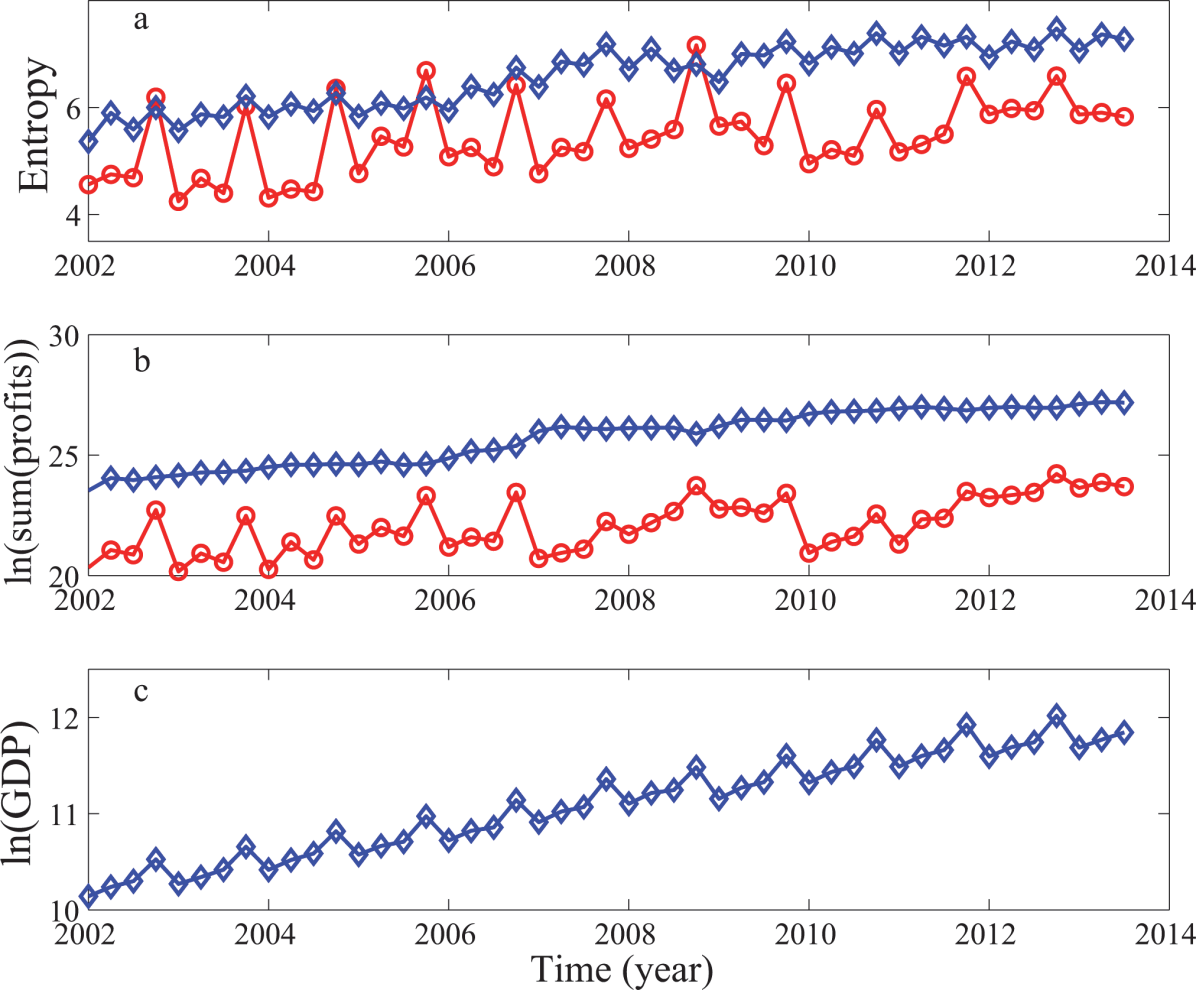
Entropy for the positive (blue) and negative (red) profits, and temporal variation of the sum of all positive (blue) and negative (red) profits, and GDP.

The rationale behind the entropy formulation lies in considering the relative strength of the two clusters formed by companies with positive and negative profits. It has been found that in normal economic times [20], the entropy for the negative profit cluster will be close to zero. Near or during recessions and crises, the entropy for the negative profit cluster will be comparable to or even larger than that for the positive profit cluster, signifying that the negative profit cluster is more stable or stronger than the positive profit cluster. Such situations can be clearly seen in China's earnings data, as shown in Fig. 1(A). In particular, we observe 4 crisis-like times in Fig. 1(A). One is at Q4 of 2008. This is clearly caused by the 2008 global financial crisis. Three others occurred in Q4 of 2002, 2004, and 2005.

Before we proceed further, we have to ask why the entropy curves have an upward trend, in particular, the trend for the curve of the positive profits almost increases monotonically? Is the trend related to the choice of the number of bins used for calculating the probabilities and then the entropy? The answer to the latter question is no. What we have observed is that after the bin-size becomes small enough, further decreasing the bin-size (or eqivalently, increasing the number of bins) only shifts the entropy curves upward, but does not alter the basic pattern presented in Fig. 1(A). We surmise that the upward trend in the entropy curves is related to the rapid growth of Chinese stock market, as well as China's GDP. To check our idea, we have shown in Fig. 1(B,C) the variation of the sum of the positive and negative profits and GDP with time (all in logarithmic scale). As expected, these curves have well-defined upward trends. In particular, the global linear trend in Fig. 1(C) has a slope almost the same as that of the entropy curve for the positive profits shown in Fig. 1(A). Therefore, the upward trends in the entropy curves are indeed at least partly due to the rapid growth of China's economy and stock market.

**Fig 2:**
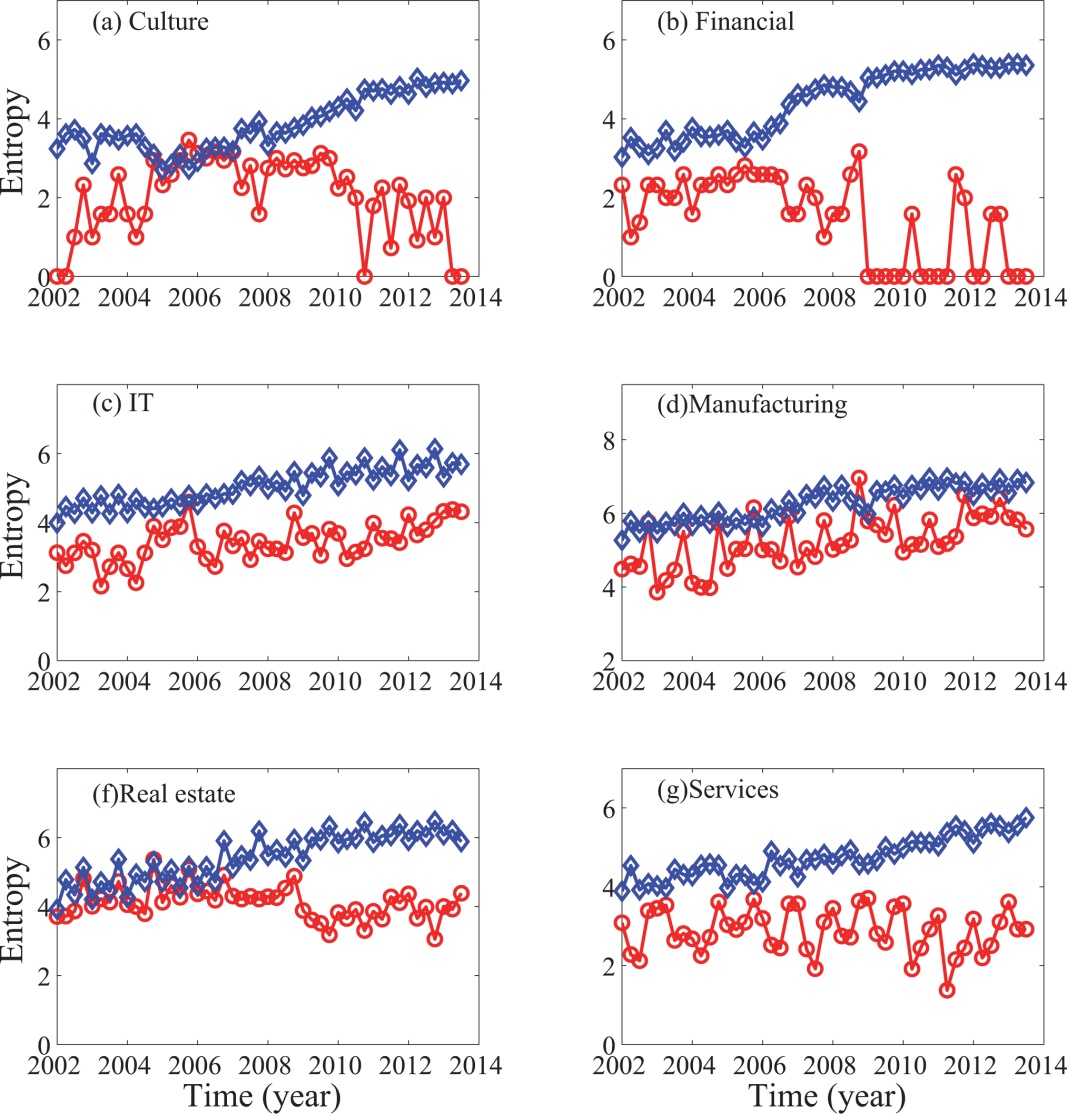
Entropy for the positive (blue) and negative (red) profits of companies listed in 6 sectors of Chinese A-share stock market.

More insights into the crisis-like behavior can be gained by examining companies in their respective sectors, an approach suggestive of an anatomy of an economy [19]. For the data analyzed here, the results are shown in Fig. 2. We observe that the sectors of culture, real estate, and manufacturing have all exhibited crisis-like behavior, with manufacturing being the worst. The situations for other sectors are clearly worse before 2007 than after 2007, albeit they did not show crisis-like behavior.

It is important to note that in the years when crisis-like behavior was not observed, Q4 was always the worst, i.e., the red curves always show a local maximum at Q4.

**Fig 3:**
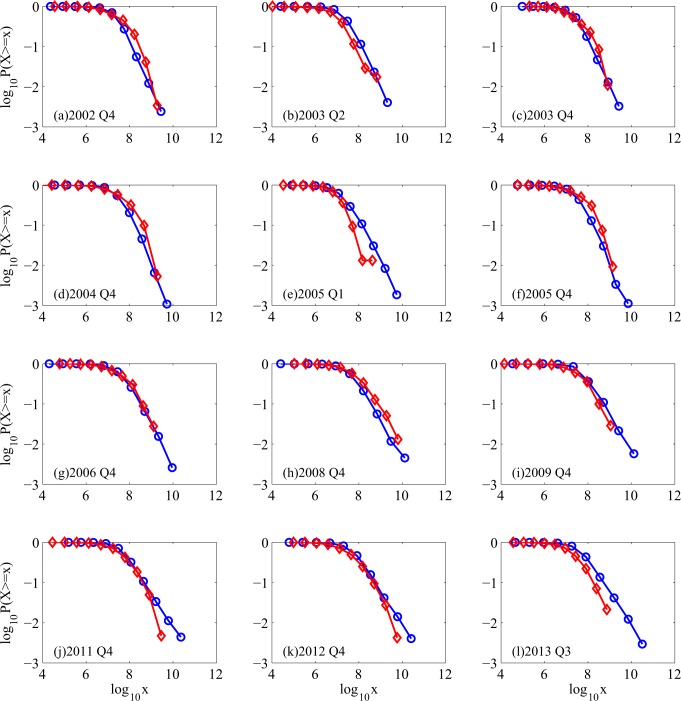
Examples of complementary cumulative distribution functions (CCDFs) in 12 different quarters.

**Fig 4:**
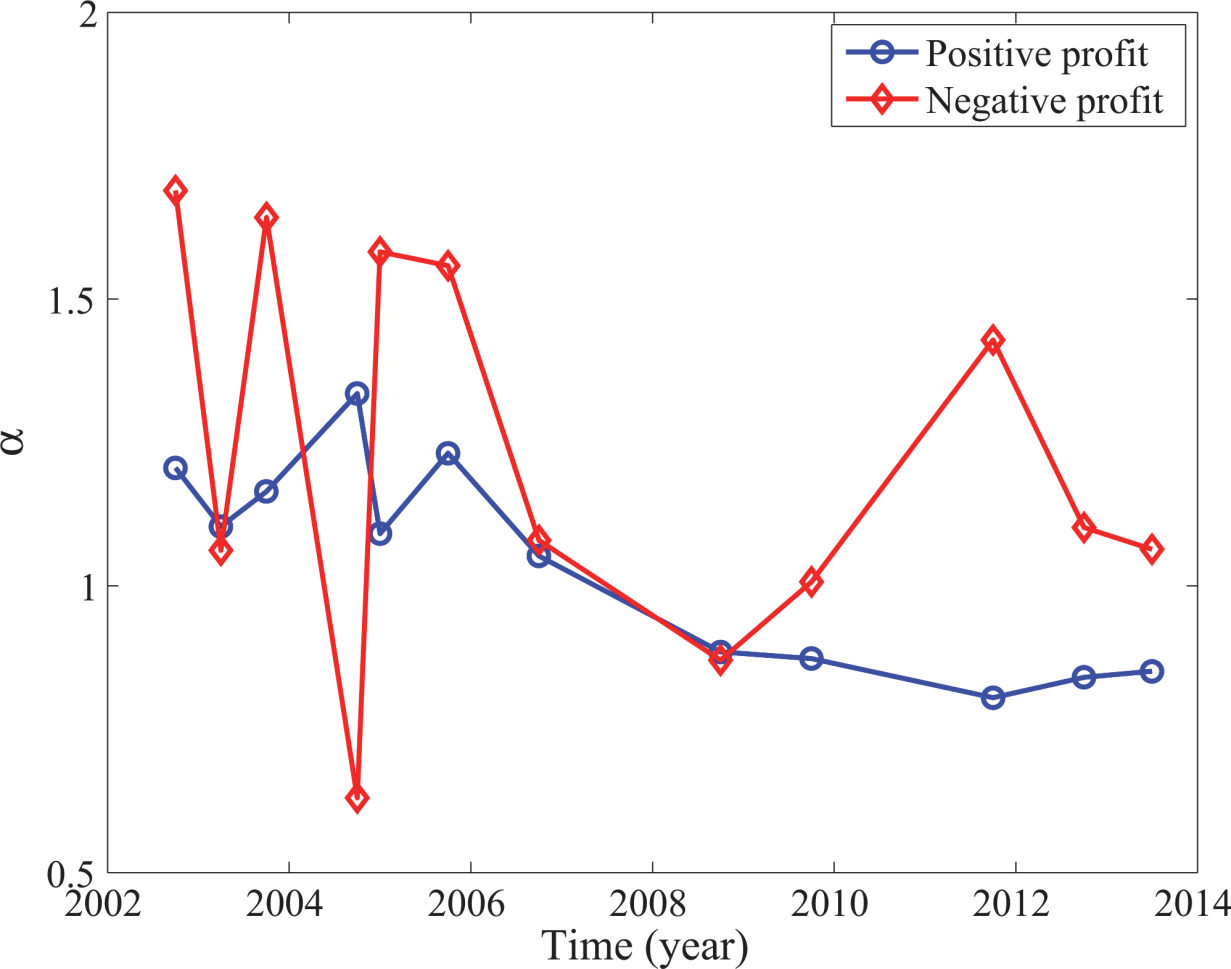
Parameter α for positive (blue) and negative (red) profits corresponding to Fig. 3.

To appreciate better the severity of the losses, we have examined the complementary cumulative distribution function (CCDF) of both the losses and the profits. Examples for a number of quarters are shown in Fig. 3. While it is not surprising that positive profits follow a Pareto law, we have observed that negative profits may also follow a Pareto law, with a well-defined α, whose values, together with those for the positive profits are shown in Fig. 4. Often due to limited scaling region for the CCDF of negative profits, the estimated α values there may not reflect the "heaviness" of the CCDF of negative profits. To be consistent with entropy values, when the CCDF curve for the negative profit is basically on top of that for the positive profits, we have to say that the CCDF for the negative profits is heavier than that for positive profits. This is precisely why the Q4 of 2002, 2004, 2005, and 2008 are the problematic times—here, the CCDF for the losses are indeed on top of those for the positive profits, so do the entropy values.

### 2 Main cause of the crisis-like behavior

Why were there crisis-like behaviors among the companies that are listed in the China's stock market? The main part of the answer has much to do with accounting irregularity, noticing that in China, accounting is only audited at the end of the year, or Q4, but not in Q1-Q3. This paves the way for companies to report profits totally inconsistent with their actual earnings in Q1-Q3. In particular, to boost their stock value, a company with losses tends to report less loss in Q1-Q3, while a company with positive profit may choose to report better than actual earnings in Q1-Q3. In both cases, Q4 earning has to be much lower than the actual one, if the total yearly profit is to reflect the actual earning. This consideration highlights why recession or crisis-like behavior in China exclusively occurred in Q4 of a number of years.

To quantify the above scenarios, let us consider earnings of the 4 quarters in a particular year. Let us denote them by $P_{i},i=1,\cdots ,4$. Let us denote the mean and the standard deviation of $P_{i},i=1,2,3$ by $\bar{P}$ and STD, respectively. We then consider the variable

$$Ratio=(\bar{P}-P_{4})/STD$$ (3)

**Fig 5:**
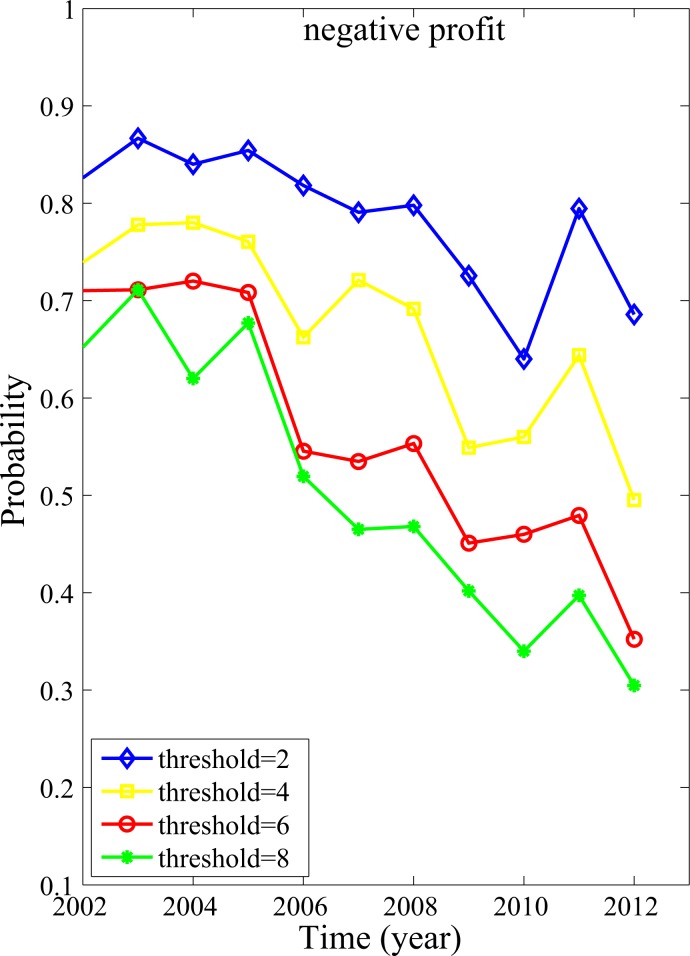
Percentage of companies with Q4 earnings satisfying $(\bar{P}-P_{4})/STD\geq threshold$ .

If $P_{i},i=1,\cdots ,4$ are drawn from a single Gaussian distribution, then it is well known that when the tail is 1, 2, and 3-standard deviations away from the mean, the tail probabilities are 32%, 5%, and 0.3%, respectively. *Ratio* defined in Equation 3 is half of the tail probability with its value equaling 1, 2, and 3, respectively. Albeit $P_{i},i=1,\cdots ,4$ may not truly follow a Gaussian distribution, it nevertheless will be instructive to consider the abundance of companies with *Ratio* exceeding certain threshold value. This abundance can be quantified by the percentage of companies with *Ration ≥ theshold* in the corresponding negative and positive profit cluster. The result for the negative profit case is shown in Fig. 5. We observe that this percentage is quite significant. In particular, the curves in Fig. 5 do not change much before 2007, for different *threshold* values. Since the threshold value can be as high as 8 while the percentage is still above 0.6, we can confidently conclude that widespread accounting irregularity had existed in China, especially before 2007 and for companies with losses, even though $P_{i},i=1,\cdots ,4$ may not follow Gaussian distribution at all. This interpretation also naturally explains why Q4 is always the worst, as we have remarked earlier.

**Fig 6:**
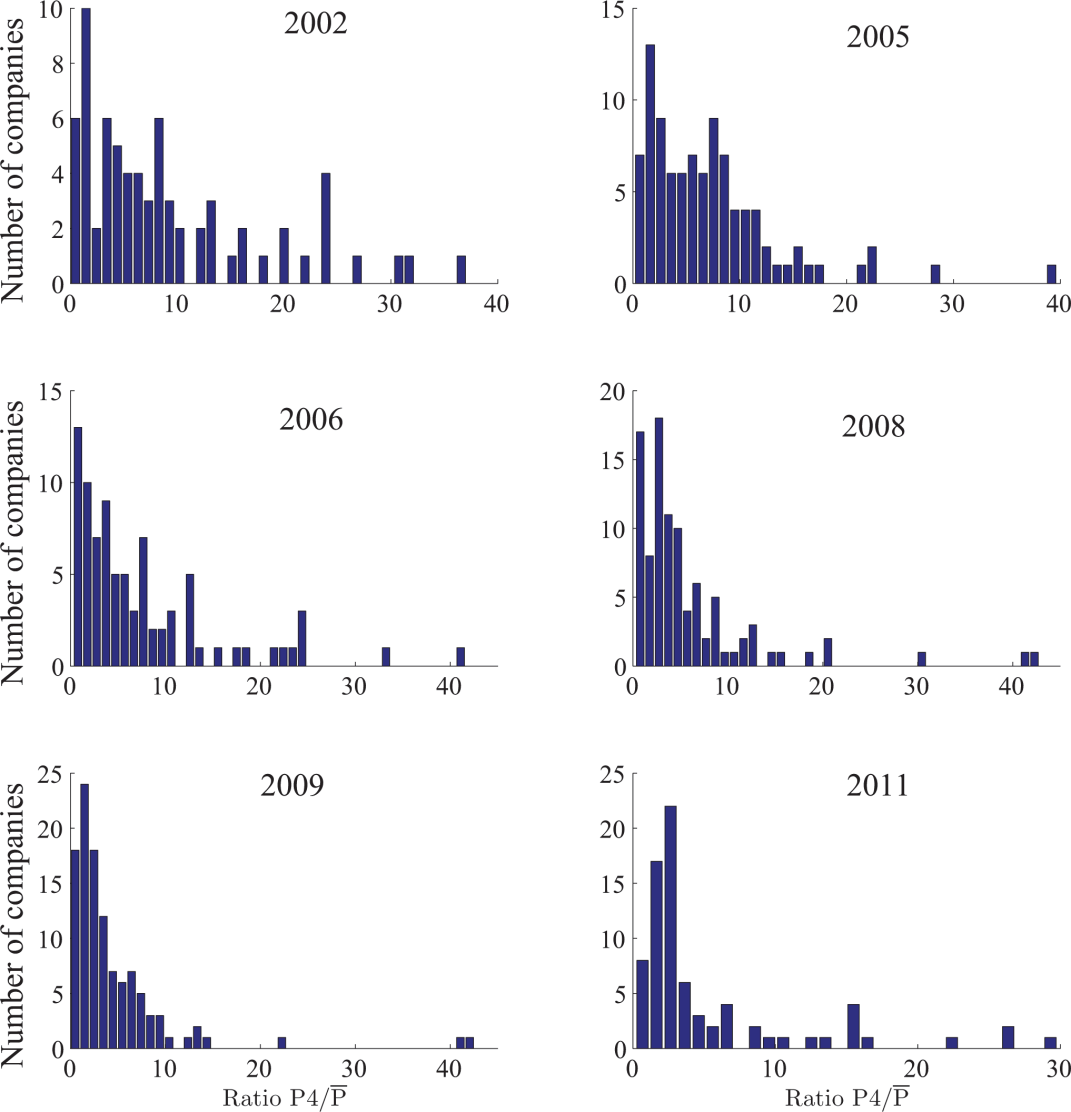
Frequency distribution for $P_{4}/\bar{P}$ .

To better see how much greater the loss in Q4 is compared with the other 3 quarters of the same year, it is instructive to evaluate the distribution of $P_{4}/\bar{P}$. Examples for 6 years are shown in Fig. 6. We observe that sometimes the reported loss in Q4 can be several tens of times that of the average loss in the first three quarters. The abnormal loss in Q4 in these and other years highlights that a number of listed companies may have engaged in profit manipulation. Indeed, a number of scenarios for profit manipulation and accounting cheating in China have been identified [24–26].

### 3 Is "big bath" a prominent factor?

To explain the abnormal Q4 loss, a number of researchers in China (see for example, [27]) have suggested that a phenomenon, called "big bath", may be a prominent factor. By "big bath", it was thought that companies with losses did not engage in profit manipulation; rather, they tended to take a "big bath", i.e., to deliberately take a huge loss in Q4 so that they can turn into profits next year. Those researchers further argued that the more losses a company had to shoulder, the more likely it would take a "big bath".

**Fig 7:**
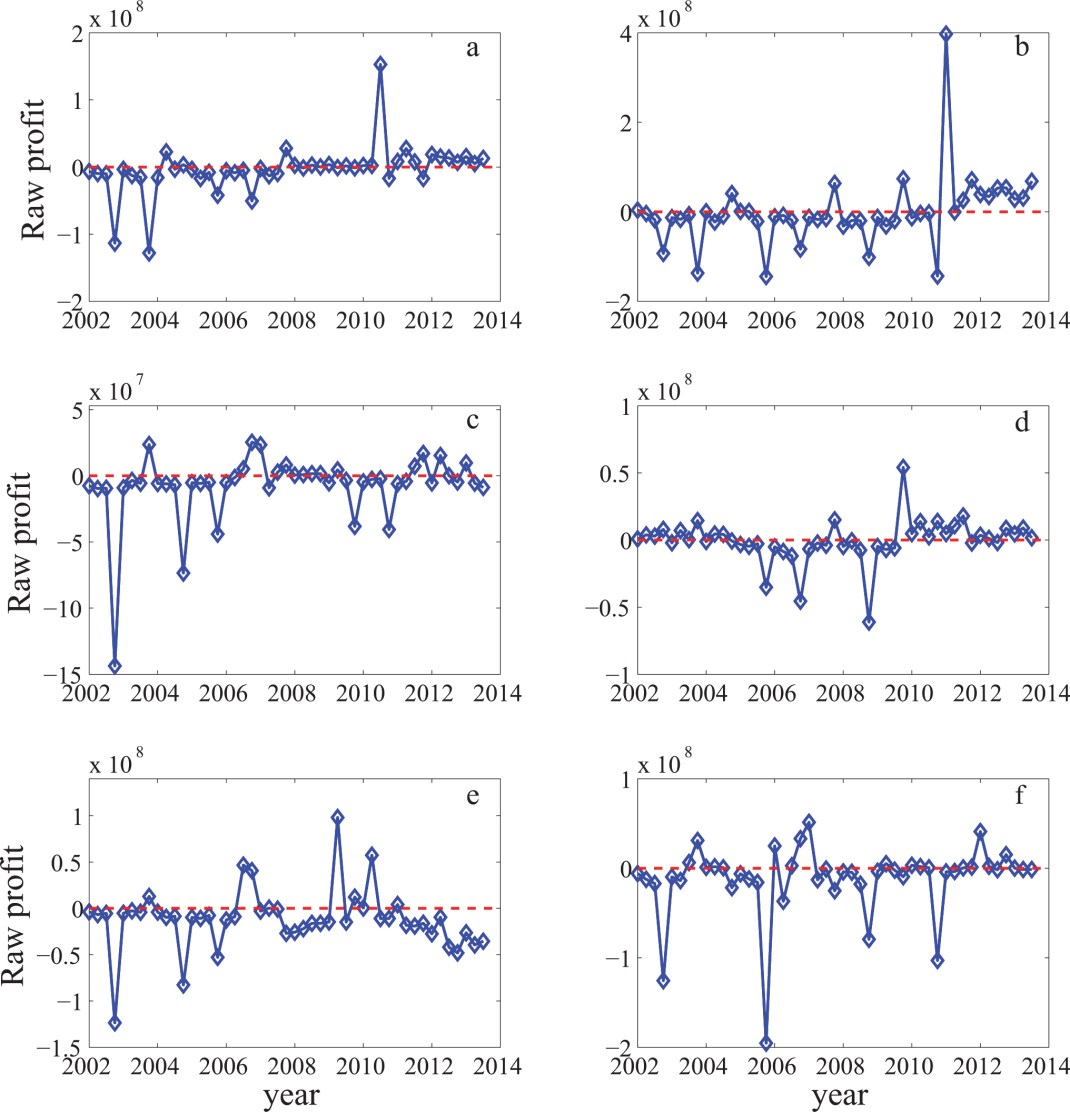
Examples of earnings data for companies with losses. The timing of local minima corresponds to Q4.

To evaluate how significant this phenomenon could be, it suffices to examine the raw earnings data of a number of companies. Fig. 7 shows data for six more or less arbitrarily chosen companies with losses. Had the "big bath" been a main factor, we should have observed impressive earnings in the years following huge Q4 losses. However, only in one year, i.e., in Fig. 7(B), had this been observed once. In all other times and for all the companies, no impressive earnings had been observed following the abnormal Q4 losses. Therefore, we have to conclude that "big bath" could at best be a secondary factor, if it was a factor at all.

## Discussion

To shed light on how healthy China's capital market is, we have examined earnings of thousands of companies listed in China's stock market. We have shown there exist economic recession or financial crisis-like behaviors in many sectors of China's economy, especially before 2007. Such behaviors exclusively occurred in the fourth quarter of a number of years. Accompanying this were the abnormally low Q4's earnings. We have identified that the latter is a vivid manifestation of widespread accounting irregularity in China before 2007—to boost stock values, many companies reported higher profits or less losses in Q1-Q3, as accounting was not audited in Q1-Q3 in China. To make the reported yearly profit more or less consistent with the actual yearly earnings, Q4's earning had to be reported much lower than the actual one, resulting in an abnormally low Q4's earning. Such behavior was clearly not consistent with a good capital market. Fortunately, such cheating has been greatly reduced, thanks to accounting reforms in China in 2007.

A few significant features highly suggest that China has been trying to converge to a market economy. One feature is related to the large impact of the 2008 global financial crisis on China's economy. The crisis-like behavior in Q4 of 2008 suggests that China's economy has been closely tied with global economy. This clearly will accelerate the rate for China to converge to a market economy. The second feature is related to the Pareto-distributed profits. Since Pareto-law is a universal law for wealth distribution, the ubiquitous Pareto-law in China's earnings data suggests that China's economy is no longer very different from other more developed countries. The third feature is still related to the Pareto-law, but for the negative profit cluster. Observing widespread losses in all these years, we have to conclude that China's economy is far from efficient. Much effort has to be made for further development.
